# Employees of the banking sector in Guizhou Province in China: prevalence of migraine, symptoms, disability and occupational risk factors

**DOI:** 10.1186/s10194-023-01591-4

**Published:** 2023-05-11

**Authors:** Du Wei, Tharani Loganathan, Li Ping Wong

**Affiliations:** 1 School of Medicine and Health Management, Guizhou Medical University, Guiyang, China; 2 Department of Social and Preventive Medicine, Faculty of Medicine, Universiti Malaya, Kuala Lumpur, Malaysia; 3 Department of Social and Preventive Medicine, Centre for Epidemiology and Evidence-Based Practice, Universiti Malaya, Kuala Lumpur, Malaysia

**Keywords:** Migraine disorder, Prevalence, Symptom assessment, Disability evaluation, Risk factors

## Abstract

**Background:**

Although studies have identified a high prevalence of migraine among employees in the banking sector, the symptoms of migraine, related disability and occupational risk factors are not well understood.

**Aims:**

To determine migraine prevalence, symptoms and disability among bank employees in Guizhou province in China and to examine occupational risk factors associated with migraine positivity and symptoms.

**Methods:**

In a cross-sectional survey, two-stage probability sampling was used to select bank employees in Guizhou province, China. From May to October 2022, uniformly trained interviewers conducted face-to-face interviews using the HARDSHIP questionnaire. Logistic regression was used to examine factors associated with migraine positivity and symptoms.

**Results:**

Of 1,985 contactable eligible subjects, 1,929 (male 45.4%, female 54.6%) completed the survey. The one-year prevalence of migraine was 27.2% (95% CI 25.2–29.2%). Of migraine-positive individuals, 11.2% had a monthly frequency ≥ 15 days, 11.8% had an attack duration > 72 h, and 14.9% had severe pain intensity. The median of days lost from work, housework and social activities due to migraine during a three-month period was 4, 3 and 2 days, respectively, with more than half (52.8%) patients reporting Grade III or IV disability. In multivariable analyses, positions in data analysis (OR = 1.8 [95% CI 1.2–2.8], *p* < 0.01) and information technology (OR = 3.8 [95% CI 1.7–8.3], *p* < 0.01) were occupational risk factors for migraine positivity. It was also found that professional positions were predictive of migraine attacks ≥ 15 days per month, administrative positions were predictive of duration > 72 h and severe pain intensity of migraine attacks, and working in remote branches was predictive of duration > 72 h.

**Conclusions:**

Migraine is prevalent among employees in the banking sector in Guizhou province in China, with a large proportion of sufferers carrying a high burden of symptoms and disability. The practical implication of this study is that the risk factors identified here could be translated to the focus of workplace monitoring and interventions to manage and prevent migraine.

**Supplementary Information:**

The online version contains supplementary material available at 10.1186/s10194-023-01591-4.

## Introduction

Migraine is one of the top ten diseases with the highest prevalence worldwide [1], and it has repeatedly been ranked as the second biggest single cause of disability by the Global Burden of Disease studies [1, 2]. Migraine is chronic and often lifelong [3], causing annoying symptoms such as pain, nausea, photophobia, phonophobia or visual aura. This disease can lead to major functional disability, affecting every aspect of a sufferer’s daily living, including work, housework and social or leisure activities. Severe migraine could be as disabling as dementia or quadriplegia and even more so than blindness, angina or rheumatoid arthritis, according to the World Health Organisation (WHO) [4]. Migraine is associated with a higher risk of cardiovascular diseases, including stroke, myocardial infarction and angina [5]. Furthermore, migraine can increase the risk of mortality from stroke, coronary heart disease and all-cause mortality by approximately 21 to 40% [6].

In China, white-collar workers are reported to have higher BMI, plasma glucose, systolic blood pressure, diastolic blood pressure, cholesterol and triglyceride levels, which put them at an elevated risk of heart diseases and stroke [7, 8]. Surveys have confirmed that white-collar workers are 1.6 times more likely to suffer from a stroke than other workers [9] and have the highest risk of cardiovascular events [10]. These health problems among white-collar workers have reached a sufficiently serious level, and they share an associated factor, migraine.

Of important note, employees in the banking sector are among the white-collar workers with a heightened risk of migraine, owing to their office environments [11], sedentariness [12, 13] and excessive job pressure [14, 15]. As such, migraines among employees in the banking sector have gained attention.

First, the office environments in banks, including the central air conditioning system, poor air quality in tight-structure offices, frequent use of photocopy machines, visual stimuli from computer screens and disturbances from temperature and noise, are major health-related complaints [16, 17]. Even with the changes in working patterns during the COVID-19 pandemic, longer screen time, which is associated with increased migraine risk [18], was observed to persist [19]. Second, it has been reported that 52.4 to 76.8% of bank employees are sedentary and never engage in physical activity [20, 21]. Moreover, the banking sector, as a result of intensive competition and economic and organisational changes, is driven to expedite digitalisation, business restructuring and service improvement [22]. So accordingly, workplace stress in the banking sector has reached a critical level, with deleterious effects on the health of employees [23]. Statistics on work-related stress by industry in the United Kingdom revealed that the banking industry ranked second out of 18 industries [24, 25].

Previous findings from the United States (U.S.) [26] and Malaysia [27] confirmed that the prevalence of migraine in the banking sector was 19.6 and 47.2%, respectively, with 1.5% of patients having symptoms for ≥ 15 days per month [27]. As a result of the agonizing symptoms of migraine [28], 39.9% of work and 38.4% of regular activities of bank employee patients were impacted [27]. Each migraine sufferer caused an annual economic loss of $1,462.4 to $1,472.9 to the banking sector in the U.S. [26] and $556 to $1,666 in Southeast Asia [27, 29].

So far as we know, empirical evidence of migraine among employees in the banking sector in China is lacking. The banking sector employs 8.6 million people, as reported by the National Bureau of Statistics of China [30]. An epidemiological study on migraine would reflect the burden of migraine in this population. Additional evidence-based knowledge of occupational risk factors for migraine would contribute to developing control and prevention programmes targeted at those who may be at elevated risk. This study intends to fill this knowledge gap by investigating migraine prevalence, symptoms (frequency, duration and intensity) and disability (as measured by the impacts on daily activities, including work, household work and family, social or leisure activities) among bank employees in China. Further, occupational risk factors associated with migraine positivity and symptoms were examined.

## Methods

### Study design and setting

This research adopted a cross-sectional design, following a standardised methodology developed by *Lifting The Burden,* a global campaign launched by an official non-profit organisation in the United Kingdom in collaboration with the WHO [28]. The Strengthening the Reporting of Observational Studies in Epidemiology (STROBE) guidelines were consulted during the reporting of our work [31].

This survey was conducted in Guizhou, a province located in southwest China. This province was chosen as the study area for two reasons. First, as an impoverished province, Guizhou’s gross domestic product (GDP) per capita ranked low among 31 provinces in mainland China over the last three years [32]. Low economic status has been demonstrated to raise concerns about migraine prevalence and attack frequency [33, 34]. Luo et al. [35] compared migraine prevalence in two neighbouring Chinese provinces and found that the province with lower economic status had a higher prevalence after controlling for climate, dietary habits and cultural norms. Second, Guizhou’s GDP growth rate outpaces that of the entire country, implying that workers in Guizhou may be experiencing high-stress levels, which are known to cause migraine.

### Participant eligibility and sample size

The inclusion criteria were that the participants were full-time employees of banks in Guizhou province in China, between the ages of 18 and 60 and in the present job for at least six months. Bank employees with a foreign nationality or who self-reported headaches caused by or related to injury, surgery, hangover, pregnancy or breastfeeding were excluded.

We calculated sample sizes for detecting migraine prevalence and for examining the association between migraine positivity and sex, ultimately selecting the largest size. The *Openepi* program and G*Power software were used. The population size of the banking sector in Guizhou is 148 thousand [30]. We referred to the parameters from Wong et al. [27] in a similar population of Malaysian banking employees and those from Yu et al. [36] in a nationally representative sample in China. To compensate for the loss of representativeness arising from the two-stage sampling of this study, the design effect was set at 3 for a conservative estimate. A 20% non-contact rate was estimated. The sample sizes calculated for detecting migraine prevalence and for examining the association between migraine positivity and sex were a minimum of 488 and a maximum of 1,931. Finally, we conservatively adopted the result of the largest sample size of 1,931.

### Sample selection

**Fig. 1:**
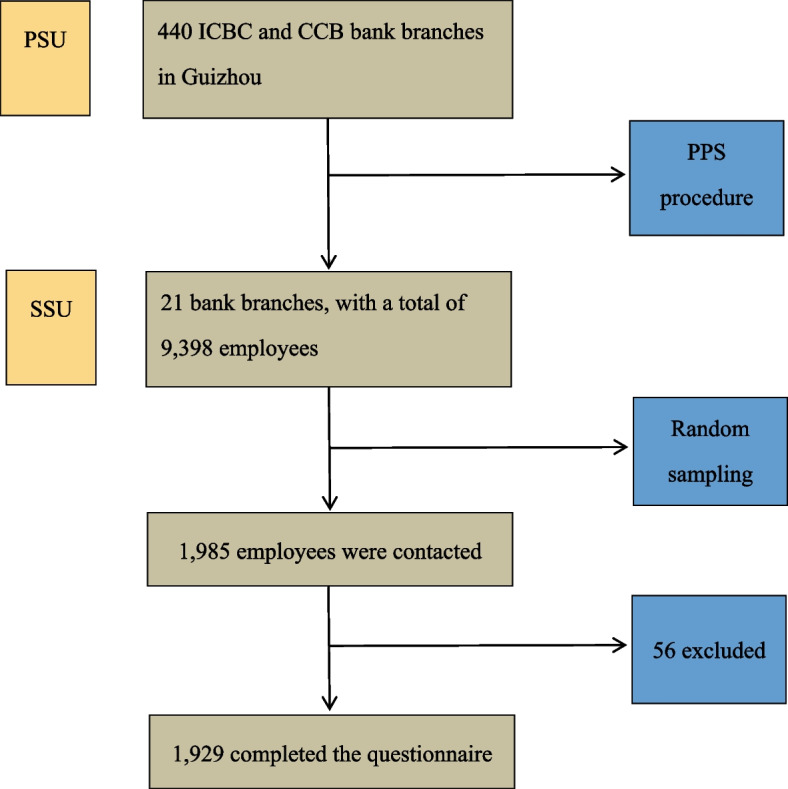
Flow diagram of the two-stage probability sampling. Abbreviations: PSU, Primary Sampling Units; SSU, Second Sampling Units; ICBC, Industrial and Commercial Bank of China; CCB, China Construction Bank; PPS, Probability Proportional to Size

Two-stage probability sampling method was utilised to select and recruit employees from banks. Participants were drawn from the first and second biggest public banks in China, the Industrial and Commercial Bank of China (ICBC) and the China Construction Bank (CCB). According to *Qichacha* (a Chinese corporate records database), as of October 2022, there were a total of 440 ICBC and CCB bank branches in Guizhou province, employing 19,919 staff. The population sizes of these bank branches ranged from 3 to 5,517. Figure 1 illustrates the flow diagram of the two-stage probability sampling.

In stage I, the Primary Sampling Units (PSU) were the 440 ICBC and CCB bank branches. Due to limited resources, 21 bank branches were selected by a probability proportional to size (PPS) procedure. In stage II, a total of 9,398 employees who were on the full-time employee lists of the 21 selected bank branches served as the Second Sampling Units (SSU). The sample size calculation indicated a minimum of 1,931 samples, giving an overall sampling rate of 20.6%. However, the participation rates varied across bank branches due to differences in individuals’ willingness to participate. A weighting procedure was implemented to ensure that each sample had an equal probability. The number of responses received from each bank branch and the weights given to the sampled subjects in each branch are displayed in Supplementary Material 1. The random selection and PPS were conducted using Microsoft Excel.

### Access and engagement of participants

From May to October 2022, interviewers distributed questionnaires face to face in selected bank branches, as migraine can be diagnosed with more sensitivity and specificity through an interview compared to a self-administered questionnaire [28]. Our interviewers, a group of uniformly trained medical students, conducted interviews as prescribed in the questionnaires’ operational manuals. The interviewers began by ascertaining whether a selected sample satisfied the eligibility criteria and subsequently obtained consent prior to inviting them to participate in the survey. To minimise information bias, interviewers clarified important questions to interviewees. Either an electronic or a printed questionnaire was provided, depending on the preferences of the invitees. The electronic questionnaire was created with *WenJuanWang*, a mobile application used extensively in China. To enhance participation, three additional attempts were made to contact those who did not respond the first time through either visits or calls without advance notification [28].

In the case of printed questionnaires, the response data were entered into a computer by two independent researchers who were blinded to each other. Any discrepancies between them were resolved by examining the original questionnaire sources.

### Instrument

The HARDSHIP questionnaire was used in this study with permission. The questionnaire comprises modules on migraine diagnosis, symptoms and disability [37]. The HARDSHIP questionnaire has been translated into Chinese language and cross-culturally validated: the migraine diagnosis module has a sensitivity and specificity of over 70%, while modules on the symptoms and disability have adequate relevance, comprehensiveness and comprehensibility [38]. This questionnaire has been used in epidemiological studies in China [36]. A positive or negative diagnosis of migraine would be determined by applying the HARDSHIP algorithm to questionnaire responses.

Migraine symptoms were investigated by monthly frequency, duration and pain intensity of attacks. Monthly frequency was ordinally categorised as ≤ 1 day, 2–7 days, 8–14 days and ≥ 15 days. To characterise duration, participants were asked about the most bothersome attacks lasting < 4 h, 4–72 h or > 72 h. Pain intensity was categrised as mild, moderate and severe for the migraine attacks that bothered participants the most.

The Headache-Attributed Lost Time (HALT) indices were imported by the HARDSHIP questionnaire to evaluate migraine-related disability [39]. The indices measure sick leave days, workdays when respondents completed less than half of their usual volume of work, missed housework days, housework days when respondents completed less than half of their usual volume of housework and missed days on family, social or leisure occasions attributable to migraine during the preceding three months. According to its developers [40], the sum of these lost days was grouped into four grades of disability: Grade I (0–5 days, minimal), Grade II (6–10 days, mild), Grade III (11–19 days, moderate) and Grade IV (≥ 20 days, severe). Grade III or IV signifies a high need for treatment [39].

### Data analysis

Data processing and analysis were performed by using SPSS software version 21 (IBM Corporation, Armonk, NY, USA). After the weighting procedure, sample characteristics were described by frequencies (N) and percentages (%). The International Classification of Headache Disorders (ICHD) criteria make a diagnosis in the following order: migraine, tension-type headaches, probable migraine, probable tension-type headaches, allowing for the separation between ‘migraine’ and ‘probable migraine’ due to the uncertainty of a questionnaire-based diagnosis. Accordingly, we presented both of them and ‘all migraine’ (the two combined) over a prevalence time frame of one year, together with their respective 95% confidence intervals (CIs). Histograms were used to depict the distributions of symptoms and disability. The continuous data on the HALT indices were checked for normality, using multiple methods, including visual inspection of histograms, QQ and PP plots, accompanied by the Shapiro–Wilk or Kolmogorov–Smirnov test. We described the impacts of migraine on daily activities by calculating the mean ± standard deviation (SD) of lost days, as well as the median and the interquartile range (IQR) in cases of non-normal distributions.

Chi-square (or Fisher’s exact when needed) tests were used to compare migraine prevalence and symptoms across different socio-demographic groups. Before conducting multivariable analyses, the data were checked for compliance with the assumptions of logistic regression. All socio-demographic variables, regardless of whether they were significant in univariate analyses, in epidemiological studies were entered into multivariable analyses using a forced-entry approach [41] rather than using *p*-values as criteria [31]. A binomial logistic regression model was run to examine factors associated with migraine positivity. Using ordinal data on symptoms, multinomial logistic regression models were employed to investigate factors associated with the frequency, duration and intensity of migraine attacks, with the lowest coded categories as the reference groups.

## Results

### Socio-demographic characteristics

We selected 2,640 potentially eligible bank employees. Subsequently, 1,985 subjects were contacted, their eligibility was confirmed, and they consented to participate. Of them, 56 questionnaires were either non-completed or inappropriately completed. Questionnaires were completed by 1,929 respondents without missing data. The participation rate was 97.2%.

The socio-demographic characteristics of the participants are displayed in Table 1. Males and females represented 45.4 and 54.6% of the sample, respectively; there was a roughly even distribution between the ages of 18 and 60. Based on the population data from *Qichacha* (a Chinese corporate records database), the female proportion of ICBC and CCB in Guizhou province (55.4%) was comparable to that of the participants in this study, albeit slightly higher. The vast majority worked in urban bank branches (86.6%) and held a bachelor’s degree or above (80.2%). Their positions were mostly in marketing and loan services (43.7%), followed by general affairs (19.8%) and cash operation (19.1%).

**Table 1:** Socio-demographics and their associations with a migraine-positive diagnosis

Characteristics	Weighted N (%) ^a^	Migraine diagnosis		Chi-square (*p*-value)	Multivariable analysis OR (95% CI)
		**Positive N (%)**^**a**^	**Negative N (%)**^**a**^		
**Total**	1929 (100)	525 (27.2)	1404 (72.8)		
**Age (years)**				26.6 (0.000)***	
18–29	428 (22.2)	80 (18.7)	348 (81.3)		Reference
30–39	465 (24.1)	157 (33.8)	307 (66.2)		2.2 (1.6–3.1)***
40–49	529 (27.4)	152 (28.7)	378 (71.3)		2.0 (1.4–2.8)***
50–60	507 (26.3)	136 (26.8)	371 (73.2)		2.0 (1.4–2.8)***
**Sex**				4.2 (0.041)*	
Male	876 (45.4)	219 (25.0)	654 (75.0)		Reference
Female	1053 (54.6)	307 (29.2)	746 (70.9)		1.3 (1.0–1.6)*
**Education**				15.7 (0.001)**	
Senior Secondary School or below	119 (6.2)	24 (20.3)	94 (79.7)		Reference
College	263 (13.6)	57 (21.7)	206 (78.3)		1.1 (0.6–1.9)
University	1379 (71.5)	381 (27.6)	999 (72.4)		1.5 (0.9–2.4)
Graduate or above	168 (8.7)	63 (37.3)	106 (62.7)		2.2 (1.2–4.0)*
**Position**^**b**^				29.4 (0.000)***	
Cash operation	369 (19.1)	95 (25.8)	273 (74.2)		Reference
Marketing and loan services	843 (43.7)	195 (23.1)	648 (76.9)		0.9 (0.7–1.2)
General affairs	381 (19.8)	115 (30.2)	266 (69.8)		1.2 (0.9–1.7)
Data analysis	132 (6.8)	51 (38.4)	82 (61.6)		1.8 (1.2–2.8)**
Finance	121 (6.3)	41 (33.9)	80 (66.1)		1.5 (0.9–2.3)
Management	35 (1.8)	8 (22.9)	27 (77.1)		0.9 (0.4–2.1)
Information technology (IT)	29 (1.5)	15 (51.7)	14 (48.3)		3.8 (1.7–8.3)**
Risk control	19 (1.0)	5 (25.0)	15 (75.0)		0.9 (0.3–2.8)
**Location of work**				1.3 (0.248)	
Urban	1671 (86.6)	317 (25.6)	923 (74.4)		Reference
Rural	258 (13.4)	208 (30.2)	481 (69.8)		1.3 (1.0–1.8)
**Relationship status**				4.8 (0.029)*	
Married or in a relationship	1240 (64.3)	317 (25.6)	923 (74.4)		Reference
Not in a relationship	689 (35.7)	208 (30.2)	481 (69.8)		1.4 (1.1–1.8)**
**Monthly salary (¥)**				10.7 (0.013)*	
Under 5,000	417 (21.6)	89 (21.3)	328 (78.7)		Reference
5,001–8,000	726 (37.7)	210 (28.9)	516 (71.1)		1.4 (1.0–1.9)*
8,001–11,000	440 (22.8)	134 (30.5)	306 (69.5)		1.4 (1.0–2.0)*
Above 11,001	346 (17.9)	52 (26.6)	147 (73.4)		1.2 (0.8–1.7)

*Abbreviations*: *N* Number, *OR* Odds Ratio, *CI* Confidence Interval

Significance level of 0.05; * *p* < 0.05; ** *p* < 0.01; *** *p* < 0.001

^a^Weighted number and percentage

^b^The position classification in this study is different from the International Standard Classification of Occupations (ISCO). This is because the ISCO comprises 10 major groups (e.g., legislators, skilled agricultural, forestry and fishery workers, and craft and related trade workers), making the ISCO more suitable for large surveys or censuses and difficult to apply directly to industry-specific studies. To be prudent, we had consulted three human resources managers of banks prior to our survey on the content validity of the position classification, and they made suggestions and ultimately endorsed its relevance and comprehensiveness

### Migraine prevalence and risk factors for migraine positivity

A total of 525 individuals were diagnosed positive for migraine. The one-year prevalence of 'all migraine' was 27.2% (95% CI 25.2–29.2%), with a migraine prevalence of 6.4% (95% CI 5.3–7.5%) and a probable migraine prevalence of 20.8% (95% CI 19.0–22.6%).

The multivariable analysis demonstrated that age, sex, education, position, relationship status and monthly salary were significant predictors of a positive diagnosis of migraine. As shown in Table 1, it was discovered that information technology (IT) positions were the most influential factor on the risk of migraine (OR = 3.8 [95%CI 1.7–8.3], *p* < 0.01), while data analysis positions were also associated with higher odds of migraine (OR = 1.8 [95%CI 1.2–2.8], *p* < 0.01), as compared to cash operation positions. Females had 1.3 (95% CI 1.0–1.6, *p* < 0.05) times higher odds of being diagnosed with this disease. The prevalence of migraine peaked among 30–39 year olds (prevalence 33.8%), followed by 40–49 year olds (prevalence 28.7%), 50–60 year olds (prevalence 26.8%) and 18–29 year olds (prevalence 18.7%). Individuals aged 30–39 (OR = 2.2 [95% CI 1.6–3.1], *p* < 0.001), 40–49 (OR = 2.0 [95% CI 1.4–2.8], *p* < 0.001) and 50–60 (OR = 2.0 [95% CI 1.4–2.8], *p* < 0.001) were more likely to be diagnosed with migraine than those aged 18–29 years. Individuals with a graduate degree or higher were more likely to be diagnosed with this disease than those with a high school degree or lower (OR = 2.2 [95%CI 1.2–4.0], *p* < 0.05). Other significant risk factors associated with migraine positivity included marriage or being in a relationship (OR = 1.4 [95% CI 1.1–1.8], *p* < 0.01), incomes between ¥5,001 and ¥8,000 (OR = 1.4 [95%CI 1.0–1.9], *p* < 0.05) and incomes between ¥8,001 and ¥11,000 (OR = 1.4 [95%CI 1.0–2.0], *p* < 0.05).

### Migraine symptoms and disability

**Fig. 2:**
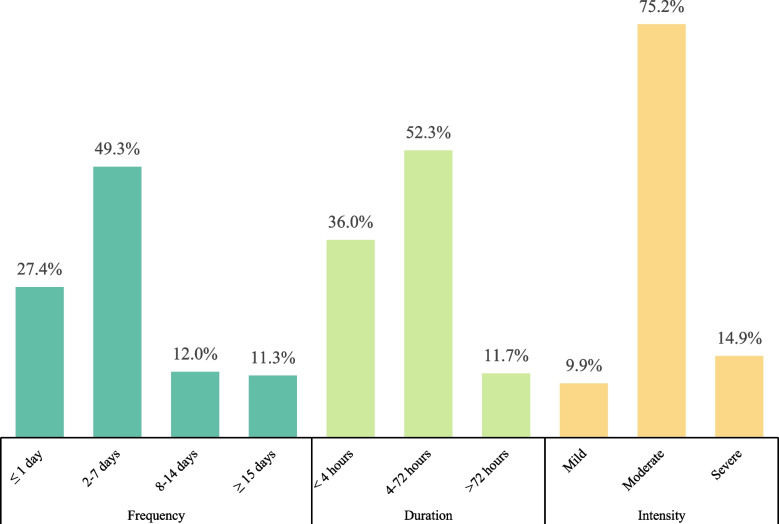
Symptoms among migraine-positive respondents

Figure 2 illustrates the distributions of the frequency, duration and intensity of attacks among migraine-positive respondents. The current study found that most of them had a monthly frequency of 2–7 days (49.3%), a duration of 4–72 h (52.4%) and moderate pain intensity (75.2%). The patients in the most severe symptom categories are of particular concern: 11.2% experienced a monthly frequency ≥ 15 days, 11.8% had attacks lasting > 72 h, and 14.9% suffered from severe pain intensity.

**Fig. 3:**
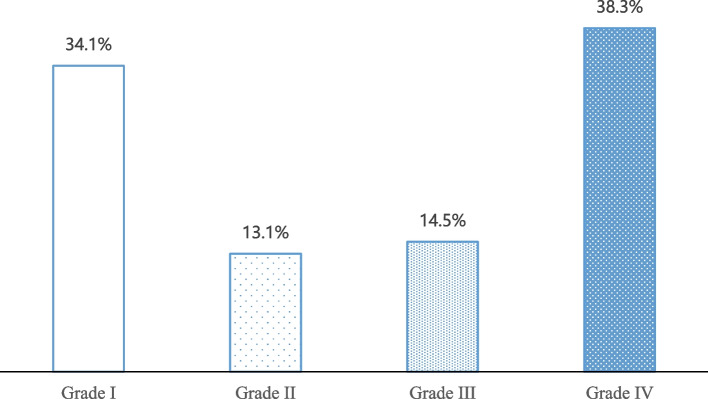
Disability grades among migraine-positive respondents

As shown in Fig. 3, of the 525 individuals diagnosed with migraine, 14.5% reported moderate disability (Grade III), and 38.3% reported severe disability (Grade IV). Based on the interpretation of the HALT indices, 52.8% of migraine sufferers (in Grade III or Grade IV) had a high treatment need.

The impacts of migraine on activities of daily living were captured, as indicated in Table 2. The distributions of these lost days were skewed. During the past three months, the median number of days lost from work, housework and social events due to migraine were 4, 3 and 2, respectively. The number of days lost from work or housework was calculated as the sum of missed days and days when productivity was reduced by more than half of the usual total. Stewart et al. [40] proved that this calculation could compensate for neglecting the number of days when productivity was reduced by less than half of the usual total.

**Table 2:** Impacts of migraine on daily activities during the last three months

Activities of daily living	Lost days attributable to migraine (days)		
	**Range**	**Mean (SD)**	**Median (IQR)**
Work	0–90	12.4 (20.8)	4 (11)
Housework	0–90	13 (22.6)	3 (13)
Family, social or leisure events	0–90	8.1 (15.8)	2 (7)

*Abbreviations*: *SD* Standard Deviation, *IQR* InterQuartile Range

### Occupational risk factors for migraine symptoms

Table 3 shows the results of the multinomial logistic regression for the frequency, duration and intensity of migraine attacks. After controlling for other factors, employees in professional positions were more likely to experience a monthly frequency of ≥ 15 days, while those in administrative positions were more likely to have attacks lasting > 72 h and of severe pain intensity. The location of work was also associated with the duration of migraine attacks, whereby those working in rural branches had a greater likelihood than their counterparts working in urban branches to suffer from attacks lasting > 72 h over lasting < 4 h.

**Table 3:** Migraine symptoms in relation to socio-demographics

Socio-demographics	Frequency			Duration		Intensity	
	**2–7 d (vs. ≤ 1 d)** **OR (95% CI)**	**8–14 d (vs. ≤ 1 d)** **OR (95% CI)**	** ≥ 15 d (vs. ≤ 1 d)** **OR (95% CI)**	**4–72 h (vs. < 4 h)** **OR (95% CI)**	** > 72 h (vs. < 4 h)** **OR (95% CI)**	**Moderate (vs. mild)** **OR (95% CI)**	**Severe (vs. mild)** **OR (95% CI)**
**Age**							
18–29	Reference	Reference	Reference	Reference	Reference	Reference	Reference
30–39	0.7 (0.3–1.5)	0.5 (0.2–1.4)	0.6 (0.2–1.5)	1.4 (0.8–2.6)	1.6 (0.6–4.4)	1.0 (0.3–2.8)	1.5 (0.4–5.5)
40–49	0.6 (0.3–1.4)	0.6 (0.2–1.7)	0.4 (0.1–1.0)	2.1 (1.1–3.9)*	1.6 (0.5–4.7)	0.9 (0.3–2.8)	1.6 (0.4–6.0)
50–60	0.4 (0.2–0.8)*	0.5 (0.2–1.6)	0.2 (0.1–0.6)**	2.9 (1.4–5.9)**	7.9 (2.6–23.7)***	0.8 (0.3–2.5)	1.2 (0.3–5.0)
**Sex**							
Male	Reference	Reference	Reference	Reference	Reference	Reference	Reference
Female	1.0 (0.7–1.6)	0.8 (0.4–1.4)	0.7 (0.3–1.2)	1.1 (0.7–1.6)	0.9 (0.5–1.6)	1.2 (0.7–2.2)	1.4 (0.7–2.9)
**Education**							
College or below	Reference	Reference	Reference	Reference	Reference	Reference	Reference
University	0.3 (0.2–0.7)**	0.4 (0.1–0.9)*	0.2 (0.1–0.6)**	0.7(0.4–1.3)	1.6 (0.6–4.0)	0.7 (0.3–1.8)	0.6 (0.2–1.7)
Graduate or above	0.6 (0.2–1.5)	0.5 (0.1–2.0)	0.7 (0.2–2.5)	0.7 (0.3–1.6)	2.6 (0.7–9.5)	1.4 (0.4–5.7)	1.1 (0.2–5.5)
**Position**^**a**^							
Cash operation	Reference	Reference	Reference	Reference	Reference	Reference	Reference
Marketing and loan services	1.0 (0.6–1.8)	2.2 (0.8–5.9)	2.7 (1.0–7.4)	0.9 (0.5–1.5)	2.8 (0.9–8.2)	1.7 (0.8–3.6)	2.3 (0.8–6.3)
Administration	1.0 (0.6–1.8)	1.7 (0.6–4.7)	2.5 (0.9–7.0)	1.2 (0.7–2.2)	5.8 (1.9–17.3)**	1.1 (0.5–2.6)	3.1 (1.1–8.8)*
Professionals	1.1 (0.5–2.5)	3.3 (1.0–10.7)	3.4 (1.0–11.5)*	1.2 (0.6–2.5)	1.7 (0.4–7.4)	2.0 (0.7–5.9)	1.2 (0.3–5.4)
**Location of work**							
Urban	Reference	Reference	Reference	Reference	Reference	Reference	Reference
Rural	0.8 (0.4–1.4)	1.4 (0.6–3.2)	1.1 (0.4–2.6)	1.3 (0.7–2.4)	2.4 (1.1–5.5)*	1.2 (0.5–3.1)	1.2 (0.4–3.6)
**Relationship status**							
Married or in a relationship	Reference	Reference	Reference	Reference	Reference	Reference	Reference
Not in a relationship	1.3 (0.8–2.0)	2.0 (1.0–3.8)*	1.1 (0.5–2.2)	1.4 (0.9–2.2)	2.4 (1.2–4.6)*	1.9 (0.9–3.8)	1.3 (0.6–3.1)
**Monthly salary (¥)**							
Under 5,000	Reference	Reference	Reference	Reference	Reference	Reference	Reference
5001–8,000	1.1 (0.6–2.1)	2.2 (0.8–5.5)	0.7 (0.3–1.7)	1.8 (1.0–3.2)*	2.4 (1.0–6.0)	0.8 (0.3–2.1)	0.4 (0.1–1.3)
8001–11,000	1.2 (0.6–2.5)	1.4 (0.5–4.0)	0.6 (0.2–1.6)	2.1 (1.1–3.8)*	1.9 (0.7–5.4)	0.5 (0.2–1.4)	0.4 (0.1–1.5)
Above 11,001	0.9 (0.4–1.9)	0.5 (0.1–2.0)	0.7 (0.2–2.0)	2.0 (1.0–3.9)	2.3 (0.7–7.3)	0.7 (0.2–2.4)	0.8 (0.2–3.0)

*Abbreviations*: *d* day, *h* hour, *OR* Odds Ratio, *IT* Information Technology

Significance level of 0.05; * *p* < 0.05; ** *p* < 0.01; *** *p* < 0.001

^a^Somes positions were combined into one group because of the presence of expected counts below 5 in the Chi-square tests. Positions in data analysis and IT were combined into professional positions, and positions in general affairs, finance, management and risk control were combined into administrative positions

## Discussion

### Findings of this study

To our best knowledge, this work is the first on migraine among employees of the banking sector in China. Of note, nearly 30% of bank employees in this study were diagnosed with migraine, which is higher than the previous nationwide estimate of 9.3% [36] among the general population using the same diagnostic tool as our study. Another smaller-scale study in China also found a lower prevalence (7.5%) than ours [38]. The higher prevalence of migraine in our study supports the two hypothesised mechanisms mentioned above. The first is that both being in the banking sector and the rapid economic growth of Guizhou province expose employees to tremendous stress, which could potentially trigger migraine. A high level of work-related stress in China’s banking sector has been found in various literature [42, 43]. Similarly, it has been well demonstrated that rapid economic growth often corresponds with heightened workplace stress due to overtime work and increased productivity [44]. The second mechanism is the well-established association between low socio-economic status and migraine [33]. In this case, Guizhou is a relatively undeveloped province, with the fourth lowest GDP per capita among 31 provinces in China in 2022 [32], which therefore is a predispose factor for migraine. The identification of migraine prevalence in the current study is crucial in helping draw the attention of Chinese policymakers to the burden of migraine in the banking sector in Guizhou province, and thus is also crucial for management efforts. However, to our best knowledge, the diagnosis and treatment of migraine, specifically in the banking sector in Guizhou province in China, have not been reported. Future studies should therefore explore the diagnosis and treatment of migraine in order to provide insights into the development of therapeutic programmes that are specific to the clinical characteristics, preferences and needs of this population. 

Migraine symptoms, including frequency, duration and intensity, were documented in our study. Existing studies concentrate on the frequency of migraine, as it is an important diagnostic criterion for chronic migraine. Despite this, it may be true that a diagnosis of chronic migraine is ‘probable chronic migraine’ using a questionnaire based on recall at a particular moment in time. The ICHD criteria acknowledge diagnostic difficulty when headaches occur ≥ 15 days per month, because their characteristics usually vary not only from day to day but also within the same day [37, 45]. However, even so, we have made efforts to identify the groups of migraine patients in the most severe symptom categories, with 11.2% experiencing a frequency ≥ 15 days per month, 11.8% having attacks lasting > 72 h and 14.9% suffering from severe pain intensity. These data show evidence of the burden of the most severe migraine symptoms among banking employees in Guizhou, China. In fact, this burden is largely avoidable, since effective and cost-effective treatments already exist [3]. Patient education should be implemented on appropriate treatments for migraine.

As a consequence of migraine symptoms, 52.8% of patients in the banking sector in Guizhou province reported Grade III or IV disability, which is higher than the 38% recorded nationally in China using the same HALT indices as in this study [36]. This demonstrates a higher burden of migraine disability in employees in the banking sector in Guizhou, compared to the general population in China. When one has a disability of Grade III or IV, it denotes that his daily activities are impacted by migraine for over 11 days in a three-month period. Not only disabled migraine patients themselves but also their families, friends, colleagues and employers were seriously affected; in the extreme cases we surveyed, migraine prevented them to continue with any work, housework and social activities. Grade III or IV disability is an indication of an urgent need for health care [39]. From a societal perspective, although the identification of migraine may lead to increased healthcare utilisation and increased direct costs, this will most likely be offset or outweighed by savings in indirect costs [46]. According to one European study, the indirect costs of migraines can be up to 13 times that of the direct costs [47]. The economic burden of migraines among employees in the banking sector in China, remains unknown, and future research is warranted to inform policymakers of the potential economic savings from workplace accommodations and interventions to reduce the burden of migraine [48, 49].

The sex and age disparities in migraine prevalence in the present study are in line with previous evidence [50, 51]: migraine influences more females than males and peaks in mid-adulthood. According to the review that integrated multiple animal studies and clinical trials [50], this sex disparity is caused by sex hormones and menstruation. However, not much is known regarding the pathophysiology of migraine evolution according to the phases of life [52]. The current study also found a graduate degree or higher to be a risk factor for migraine, which is in agreement with previous observations [53]. An important finding is that positions in data analysis and IT in banks were associated with a higher risk of migraine, as compared to cash operation positions. The association between professional positions and higher migraine prevalence has been previously reported [54, 55] and, in particular, there are epidemiological studies focusing on IT employees with migraine [56, 57]. These occupational risk factors may be related, at least in part, to a long duration of screen time exposure. Staring at screens for a long time, such as those on smartphones or computers, has been observed to increase migraine risk [18, 58]. Nevertheless, it remains a challenge for interventions for screen time in the banking sector, as the majority of employees spend long hours on computers for work. According to the literature, workplace intervention strategies proposed by researchers include changing organisational social norms so that taking a break or leaving one’s desk does not mean unproductivity [59], using a webcam to obtain data for health monitoring such as blink rate [60], and promoting worksite physical activity to reduce screen time [61]. Collectively, this study highlights the necessity for sex-, age-, education- and occupation-based strategies (interventions targeted at specific demographic populations) for migraine management and prevention in the workplace.

Further, to our best knowledge, this work is the first to uncover occupational predictors of migraine symptoms. Understanding what aggravates migraine symptoms is a critical public health priority. Looking at migraine frequency as an example, chronic migraine patients suffer more debility and prostration, as well as more physical and psychiatric comorbidities, a substantially higher disability, and thus lower quality of life, when compared to episodic migraine patients [62]. This study found that professional positions were predictive of migraine attacks ≥ 15 days per month. Job positions can be a very important screening tool for the early identification of patients with high-frequency migraines who warrant immediate intervention. Our findings suggest that in order to reduce the frequency of migraine attacks, workplace-based screening could be targeted towards those in professional positions. Of the three types of migraine symptoms, frequency has been studied most in the literature as it is an important diagnostic criterion for chronic migraine. The most common cause of high-frequency migraine is the overuse of medication [62]. One study implemented a workplace intervention that included medication education, and after a year, participants reported a 76.1% decrease in migraine frequency [63]. This indicates the feasibility and effectiveness of migraine intervention in the workplace. Apart from migraine frequency which requires special attention to medication overuse, the clinical management of the three types of migraine symptoms (frequency, intensity and duration), is similar [64]. This study found that administrative positions were predictive of duration > 72 h and severe pain intensity of migraine attacks, and working in remote branches was predictive of duration > 72 h. To reduce the pain intensity and duration, workplace-based screening could be targeted towards those in administrative positions and working in remote branches. In addition, although clinical management guidelines for migraine are available, studies show the efficacy of complementary and alternative therapies, such as physical therapy, in reducing the duration of migraine attacks [65]. To conclude, priority in future interventions should be given to individuals in professional positions to reduce the frequency of migraine attacks, to those in administrative positions to reduce the pain intensity and duration, and to those working in rural branches to reduce the duration.

### Strengths and limitations

The diagnostic tool used in our study, the HARDSHIP questionnaire, has been demonstrated to be optimal for diagnosing migraine in non-clinical settings in a wide range of cultures [66]. Moreover, the probability sampling strategy used in our study, together with the reasonable participation rate, allowed us to obtain a representative sample of bank employees in Guizhou province.

Though we did our best to study the burden of migraine and occupational risk factors among bank workers, this research is not free from limitations. First, our data came from a single province in China rather than a national sample, somewhat limiting the generalisability of our findings to a national level. Nevertheless, in this study, a representative sample of employees from the banking sector in Guizhou province was obtained, and hence the findings represent the population in Guizhou and are informative for this province. On the other hand, the findings on occupational risk factors for migraine have external validity, since the environments and exposures are largely similar among banks and other office-based organisations in China. Second, this survey did not consider other important independent variables that influence migraine, such as stress and other occupational risk factors, which limits the examination of mediating and moderating effects. The third limitation is the self-reporting bias induced by the survey research. Fourthly, this study was limited by the cross-sectional design, whereby we did not collect follow-up data, making migraine aetiology and progression impossible to track. Despite these limitations, the findings of this study provided important insights into the burden of migraine in Guizhou province in China. Future research should include the diagnostic and treatment status of migraine, as well as the economic impact, to provide more information to help decrease the burden of migraine.

## Conclusions

Migraine is prevalent among employees in the banking sector in Guizhou province in China, with a large proportion of sufferers carrying a high burden of symptoms and disability. Positions in data analysis and IT in banks are occupational risk factors for migraine positivity. Professional and administrative positions, as well as working in rural branches, are occupational risk factors for a high symptom burden of migraine. The practical implication of this study is that the risk factors identified here could be translated into the focus of workplace monitoring and interventions targeted at managing and preventing migraine.

## Supplementary Information

**Additional file 1:** **Supplementary Material 1.** List of sampled bank branches and number of respondents.

## Data Availability

The datasets generated and/or analysed during this study are available upon reasonable request from the corresponding authors.

## Abbreviations

OR: Odds Ratio
WHO: World Health Organisation
U.S.: United States
STROBE: Strengthening the Reporting of Observational Studies in Epidemiology
GDP: Gross Domestic Product
PSU: Primary Sampling Units
SSU: Second Sampling Units
ICBC: Industrial and Commercial Bank of China
CCB: China Construction Bank
PPS: Probability Proportional to Size
HALT: Headache-Attributed Lost Time
ICHD: International Classification of Headache Disorders
CI: Confidence Intervals
SD: Standard Deviation
IQR: InterQuartile Ranges
IT: Information Technology
