# Supplementary material for: Employees of the banking sector in Guizhou Province in China: prevalence of migraine, symptoms, disability and occupational risk factors
Source: J Headache Pain. 2023 May 11;24(1):52. doi: 10.1186/s10194-023-01591-4 (PMC10173247; doi:10.1186/s10194-023-01591-4)
Supplement: Supplementary file 1 — Additional file 1: Supplementary Material 1. List of sampled bank branches and number of respondents. [file 10194_2023_1591_MOESM1_ESM.docx]

**Supplementary Material 1** List of sampled bank branches and number of respondents

| **No** | **Bank branch** | **Branch size** | **Number of respondents** | **Probability of selection** | **Weight** |
| --- | --- | --- | --- | --- | --- |
| **1** | CCB1 | 5517 | 1020 | 0.0088 | 113.33 |
| **2** | CCB2 | 377 | 69 | 0.0087 | 114.48 |
| **3** | CCB3 | 377 | 66 | 0.0084 | 119.68 |
| **4** | CCB4 | 360 | 57 | 0.0076 | 132.33 |
| **5** | CCB5 | 340 | 47 | 0.0066 | 151.57 |
| **6** | CCB6 | 118 | 33 | 0.0133 | 74.92 |
| **7** | CCB7 | 29 | 13 | 0.0214 | 46.74 |
| **8** | CCB8 | 22 | 12 | 0.0260 | 38.41 |
| **9** | CCB9 | 19 | 9 | 0.0226 | 44.23 |
| **10** | CCB10 | 16 | 9 | 0.0268 | 37.25 |
| **11** | CCB11 | 13 | 9 | 0.0330 | 30.26 |
| **12** | CCB12 | 8 | 5 | 0.0298 | 33.52 |
| **13** | ICBC1 | 467 | 155 | 0.0158 | 63.13 |
| **14** | ICBC2 | 460 | 113 | 0.0117 | 85.29 |
| **15** | ICBC3 | 425 | 111 | 0.0125 | 80.22 |
| **16** | ICBC4 | 425 | 73 | 0.0082 | 121.98 |
| **17** | ICBC5 | 159 | 40 | 0.0120 | 83.29 |
| **18** | ICBC6 | 134 | 36 | 0.0128 | 77.99 |
| **19** | ICBC7 | 80 | 26 | 0.0155 | 64.47 |
| **20** | ICBC8 | 42 | 19 | 0.0216 | 46.32 |
| **21** | ICBC9 | 10 | 7 | 0.0334 | 29.93 |
| Total | | 9,398 | 1,929 |  |  |
